# The prediction model for intraoperatively acquired pressure injuries in orthopedics based on the new risk factors: a real-world prospective observational, cross-sectional study

**DOI:** 10.3389/fphys.2023.1170564

**Published:** 2023-07-21

**Authors:** Ning Li, Dalei Cui, Li Shan, Haixia Li, Xuelian Feng, Huilan Zeng, Lezhi Li

**Affiliations:** ^1^ Department of Anesthesia and Surgery, Xiangya Hospital, Central South University, Changsha, Hunan, China; ^2^ Teaching and Research Section of Clinical Nursing, Xiangya Hospital of Central South University, Changsha, China; ^3^ National Clinical Research Center for Geriatric Disorders, Xiangya Hospital, Central South University, Changsha, Hunan, China; ^4^ Xiangya School of Nursing, Central South University, Changsha, Hunan, China

**Keywords:** pressure injuries, intraoperatively acquired pressure injuries, orthopedic, risk factors, prediction model

## Abstract

**Introduction:** Orthopedic patients are at high risk for intraoperatively acquired pressure injuries (IAPI), which cause a serious issue and lead to high-expense burden in patient care. However, there are currently no clinically available scales or models to assess IAPI associated with orthopedic surgery.

**Methods:** In this real-world, prospective observational, cross-sectional study, we identified pressure injuries (PI)-related risk factors using a systematic review approach and clinical practice experience. We then prepared a real-world cohort to identify and confirm risk factors using multiple modalities. We successfully identified new risk factors while constructing a predictive model for PI in orthopedic surgery.

**Results:** We included 28 orthopedic intraoperative PI risk factors from previous studies and clinical practice. A total of 422 real-world cases were also included, and three independent risk factors—preoperative limb activity, intraoperative wetting of the compressed tissue, and duration of surgery—were successfully identified using chi-squared tests and logistic regression. Finally, the three independent risk factors were successfully used to construct a nomogram clinical prediction model with good predictive validity (area under the ROC curve = 0.77), which is expected to benefit clinical patients.

**Conclusion:** In conclusion, we successfully identified new independent risk factors for IAPI-related injury in orthopedic patients and developed a clinical prediction model to serve as an important complement to existing scales and provide additional benefits to patients. Our study also suggests that a single measure is not sufficient for the prevention of IAPI in orthopedic surgery patients and that a combination of measures may be required for the effective prevention of IAPI.

## Introduction

Pressure injuries (PI) refers to tissue breakdown and necrosis in the body due to prolonged pressure on local tissues, resulting in impaired blood circulation, lack of tissue nutrition, and loss of normal skin function (Edsberg et al., 2016). Intraoperatively acquired pressure injuries (IAPI) refers to tissue damage caused by any pressure associated with surgery, which mostly occurs 1–3 days post-surgery and may also occur up to 6 days after surgery (Nilsson, 2013; Edsberg et al., 2016). The previous study and international guidelines for pressure injuries explicitly include surgical patients as a high-risk group for pressure injuries (Gao et al., 2018; Panel. EPUA and Panel. NPIA, 2019).

Orthopedic patients are at high risk for IAPI because of the preoperative limitation of movement, the special positions they adopt during surgery, and the increased friction and shear forces caused by the use of hammers and chisels during surgery. Lin et al. (2017) found a 23.0% incidence of PI in spine surgery patients. The incidence of IAPI in orthopedic patients is higher than the average incidence of IAPI in patients undergoing comprehensive surgery, suggesting that orthopedic surgery is a risk factor for the occurrence of IAPI (Choi et al., 2021; Techanivate et al., 2021); therefore, there is an urgent need to pay attention to and take measures to manage the occurrence of IAPI in orthopedic surgery. The use of prognostic models to predict the risk of PI occurrence is recommended, and the risk assessment is noted to be the first step toward high-quality PI management. However, there are currently no clinically available scales or models to assess IAPI associated with orthopedic surgery, which cause a serious issue and lead to high-expense burden in patient care.

In this study, we identified PI-related risk factors using a systematic review approach and clinical practice experience. We then prepared a real-world, prospective observational, cross-sectional study to identify and confirm risk factors using multiple modalities. We have successfully identified new risk factors while constructing a predictive model for pressure injuries in orthopedic surgery. This study is expected to provide a reference for clinical practice in the identification of intraoperative skin pressure injuries on time and bring more benefits to patients.

## Methods

### Identification of pressure injury-related risk factors

This study was initiated with the collection of expert consensuses, clinical practice guidelines, systematic reviews, and observational studies on risk factors for IAPI in orthopedic patients through a systematic review search method (Supplementary Figure S1). A description of the search strategy is shown in the Supplementary Material. Relevant literature was analyzed, summarized, and organized comprehensively, and a pool of entries on PI risk factors for orthopedic surgery patients was formulated based on the reliability of evidence and clinical adaptability of risk factors in the literature and clinical practice experience. A total of 28 risk factors were included: age, gender, body mass index (BMI), incontinence, hypertension, history of hyperlipidemia, history of diabetes, surgical site, preoperative skin condition, preoperative limb activity, the level of serum albumin, preoperative corticosteroids, fasting time, duration of surgery, intraoperative wetting of the compressed part, surgical position, intraoperative use of the arch frame, intraoperative use of head rest and head ring, mechanical ventilation, intraoperative use of sponge pads/soft pillows, intraoperative use of fluid pads, intraoperative use of gel pads, intraoperative use of foam dressings, American Society of Anesthesiologists (ASA) grade, intraoperative use of traction beds, intraoperative use of tourniquets, intraoperative use of internal fixation devices, and intraoperative surgical bed adjustment angle (Lindgren et al., 2005; Slowikowski and Funk, 2010; Gao et al., 2018; Ueno et al., 2020; Techanivate et al., 2021).

### The Xiangya real-world cohort

A total of 422 patients who underwent orthopedic surgery at the Xiangya Hospital of Central South University from June 2022 to October 2022 were recruited for the prospective observational, cross-sectional study. Patient identification was performed according to the inclusion and exclusion criteria. The study was approved by the Ethics Institutional Committee of Xiangya School of Nursing, Central South University.

Inclusion criteria: 1. Patients undergoing orthopedic surgery; 2. permission from a medical condition; 3. no mental illness and severe cognitive impairment; and 4. signed informed consent form and participated voluntarily.

Exclusion criteria: 1. Preoperative presence of serious skin diseases or skin injuries where skin integrity could not be easily observed; 2. preoperative presence of PI; 3. postoperative prohibition of turning in accordance with medical advice and inability to observe the skin condition of patients; and 4. infants, toddlers, and preschoolers. According to the collection item pool of PI risk factors, data were collected from patients to evaluate the presence of PI and the degree of injury.

### Criteria for pressure injury classification

Adopting the PI staging criteria proposed in the Prevention and Treatment of Pressure injuries: Clinical Practice Guideline (Panel. EPUA and Panel. NPIA, 2019): Stage 1: Erythema appears and does not fade with pressure, erythema-associated changes in skin temperature, sensation, hardness, and finger pressure fading may appear in advance, but the skin tissue is intact, which does not include purple and maroon changes in the skin. Stage 2: The dermis at the wound is exposed, pink, and surrounded by wetness, with the appearance of plasmocyte blisters and partial absence of the cortex. Stage 3: The wound is visible as flesh granulation, fatty tissue, covered with rotting flesh or scorched scabs, with margins curling inward, no cartilage or bone, tendon, cruciate ligament, fascia, and muscle exposition, with subterranean or sinus tracts, and the skin is missing entirely. Stage 4: Covered by rotting flesh or scabs with sinus tracts or subterranean tracts, with exposed fascia, muscle, or bone, and the skin and tissue are missing entirely. Unstageable: The extent of the injury is covered by rotting flesh or scabs, and it is difficult to confirm the extent of tissue absence, with a total absence of skin and tissue; deep tissue injury: localized skin is intact or broken, showing persistent finger pressure without discoloration, or epidermal separation of the wound bed showing black or congested blisters. The assessment of IAPI and the measures of covariates were performed by two independent researchers (LN and SL). When the results were inconsistent, the ascertainment was confirmed by a third independent researcher (CDL). The assessment was conducted twice: (i) before entering the operating room: if the patient already had a preoperative pressure injury, the patient could not be included in this study; (ii) at the end of the procedure: the occurrence of intraoperatively acquired pressure injury can be directly discerned.

### Grouping methods

To better assess the cut-off points for grouping of continuous variables, the pROC program package was used to outline the receiver operating characteristic (ROC) curves (Greiner et al., 2000) for preoperative fasting time, operative time, and serum albumin level according to the outcome of pressure injury and to obtain the optimal cut-off points. As for BMI and age, we grouped the patients according to previous studies focused on the IAPI (Gao et al., 2018; Tervo-Heikkinen et al., 2022).

### Statistical analysis

Univariate and multivariate regression analyses were performed using SPSS 25.0 and R 4.1.3 statistical software, respectively. Independent sample *t*-test, analysis of variance, and χ^2^-test were used to compare the differences in each characteristic between the two groups of orthopedic patients containing intraoperative pressure injury cases and controls. One-way unconditional logistic regression analysis was used to assess the relationship between the variables of interest and the occurrence of IAPI in orthopedic patients. Variables with statistically significant differences were subjected to multi-factor unconditional logistic regression analysis of the factors influencing IAPI in orthopedic patients, and differences were considered statistically significant at *p* < 0.05. Based on the logistic regression analysis results, a nomogram model was constructed using R 4.1.3 for predicting pressure-related injury in the operating room. The nomogram model was implemented through the rms package in R software. Based on the collected data of the patients, the patients were categorized into two cohorts, namely, the training set and the validation set, in the ratio of 7:3 (Supplementary Figure S2).

## Results

### Clinical characteristics of the Xiangya real-world cohort

A total of 422 patients who underwent orthopedic surgery from June 2022 to October 2022 at Xiangya Hospital of Central South University were recruited. Among them, 212 were men and 210 were women, aged 7–90 years. The surgical site includes joints, trunk, vertebral bones, long limbs, and pelvic bones. The most common surgical sites in orthopedics are included in Table 1. We identified 66 patients with IAPI, and all of them were in Stage 1 (Supplementary Figure S3).

**TABLE 1:** Effect of clinical baseline characteristics on the incidence of IAPI.

Risk factor		IAPI		*p*
		Yes	No	
Age	<40	23	85	0.01
	40–65	30	159	
	66–80	9	104	
	>80	4	8	
Gender	Male	41	171	0.05
	Female	25	185	
BMI	<18.5	12	38	0.25
	18.5–22.9	18	119	
	23–24.9	13	89	
	≥25	23	110	
Incontinence	Yes	0	6	0.60
	No	66	350	
Hypertension	Yes	15	102	0.01
	No	51	154	
Hyperlipidemia	Yes	5	20	0.74
	No	61	336	
Diabetes	Yes	8	38	0.90
	No	58	318	
Surgical site	Joint	4	18	0.00
	Vertebra	40	281	
	Pelvic bone	4	2	
	Long bone	18	50	
	Trunk	0	5	
Preoperative skin condition	Normal	49	279	0.14
	Wet/erythema/Philippines thin	16	77	
	Essence/edema/blossom	1	0	
Preoperative limb activity	Unlimited	29	161	0.00
	Partial restriction	27	179	
	Completely restricted	10	16	
Serum albumin level	Low	27	104	0.08
	High	39	252	
Preoperative corticosteroids	Yes	4	13	0.32
	No	62	343	
Fasting time	<20.25 h	64	353	0.18
	≥20.25 h	2	3	

### Effect of different risk factors on the incidence of IAPI

**FIGURE 1:**
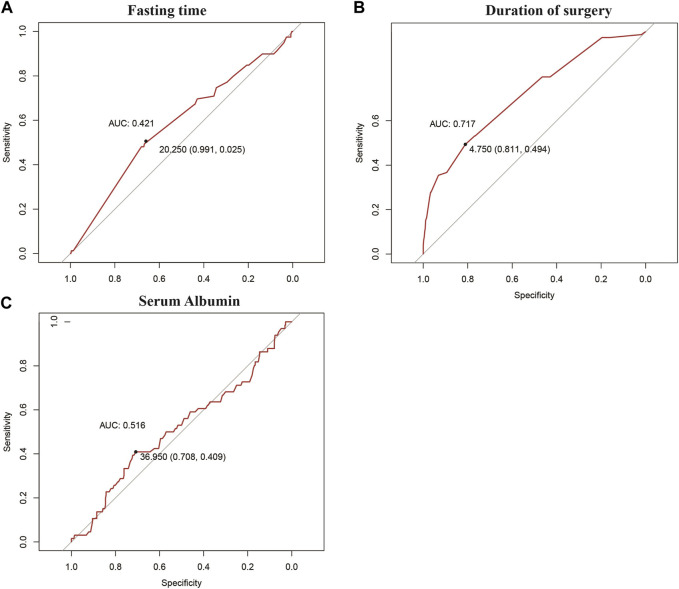
Best cut-off points obtained for grouping of continuous variables via the ROC curve: **(A)** fasting time, **(B)** duration of surgery, and **(C)** serum albumin level.

This study included 422 patients, of which 66 developed IAPI and 356 did not develop IAPI. The optimal cut-off values for preoperative fasting time and operative time were 20.250 h and 4.75 h, respectively. The most recent cut-off value for preoperative serum albumin level was 36.95 g/L (Figure 1).

The chi-squared test was used to compare the differences in the impact of risk factors on the incidence of IAPI. The results showed that the differences in age, gender, hypertension, surgical site, preoperative limb activity, duration of surgery, intraoperative wetting of the compressed part, intraoperative use of the arch frame, intraoperative use of head rest and head ring, and intraoperative surgical bed adjustment angle were statistically significant (*p* < 0.05). On the contrary, other risk factors were not statistically significant (*p* > 0.05) (Table 1; Table 2).

**TABLE 2:** Effect of intraoperative characteristics on the incidence of IAPI.

Risk factor		IAPI		*p*
		Yes	No	
Duration of surgery	<4.75 h	29	291	0.00
	≥4.75 h	37	65	
Intraoperative wetting of the compressed part	Yes	14	36	0.02
	No	52	320	
Surgical position	Prone position	36	234	0.06
	Supine position	17	89	
	Side-lying position	12	31	
	Beach chair	1	2	
Intraoperative use of the arch frame	Yes	47	293	0.05
	No	19	63	
Intraoperative use of head rest and head ring	Yes	47	294	0.05
	No	19	62	
Mechanical ventilation	Yes	60	330	0.80
	No	6	26	
Intraoperative use of sponge pads/soft pillows	Yes	33	191	0.68
	No	33	165	
Intraoperative use of fluid pads	Yes	38	159	0.07
	No	28	197	
Intraoperative use of gel pads	Yes	14	86	0.72
	No	52	270	
Intraoperative use of foam dressings	Yes	0	7	0.60
	No	66	349	
ASA grade	1	1	0	0.10
	2	15	82	
	3	48	271	
	4	2	3	
Intraoperative use of traction beds	Yes	6	33	1.00
	No	60	323	
Intraoperative use of tourniquets	Yes	4	9	0.13
	No	62	347	
Intraoperative use of internal fixation devices	Yes	60	341	0.17
	No	6	15	
Intraoperative surgical bed adjustment angle	Yes	9	17	0.01
	No	57	339	

### Relationship between different risk factors and the incidence of IAPI

In order to better explore the relationship between risk factors and IAPI, 28 risk factors were included in the univariate logistic regression analysis. The results were as follows: patients aged between 66 and 80 years had decreased risk of IAPI (OR = 0.32, 95%CI = 0.14–0.73, *p* = 0.01); women had decreased risk of IAPI (OR = 0.56, 95%CI = 0.33–0.97, *p* = 0.04); and the patients with BMI between 23 and 24.90 had decreased risk of IAPI (OR = 0.48, 95%CI = 0.21–1.08, *p* = 0.04). The pelvis had significantly increased risk of IAPI (OR = 9, 95%CI = 1.2–67.42, *p* = 0.03); the patients with completely limited limb activity in the preoperative period had significantly increased risk of IAPI (OR = 3.47, 95%CI = 1.43–8.39, *p* = 0.01); patients with surgery time ≥4.75 h had significantly increased risk of IAPI (OR = 5.71, 95%CI = 3.28–9.95, *p* < 0.001); intraoperative wetting of the compressed part showed significantly increased risk of IAPI (OR = 2.39, 95%CI = 1.21–4.74, *p* = 0.01); patients with intraoperative use of the arch frame had decreased risk of IAPI (OR = 0.53, 95%CI = 0.29–0.97, *p* = 0.04); patients with intraoperative use of head rest and head ring had decreased risk of IAPI (OR = 0.52, 95%CI = 0.29–0.95, *p* = 0.03); the adjustment angle of the surgical bed during operation showed significantly increased risk of IAPI (OR = 3.15, 95%CI = 1.34–7.41, *p* = 0.01). Furthermore, other risk factors were not statistically significant (*p* > 0.05) (Table 3; Table 4).

**TABLE 3:** Univariate logistic regression analysis of the correlation between clinical baseline characteristics and IAPI.

Risk factor	Subgroup	OR	95%CI	*p*
Age	<40	1.0		
	40–65	0.7	0.38–1.28	0.24
	66–80	0.32	0.14–0.73	0.01
	>80	1.85	0.51–6.68	0.35
Gender	Female	0.56	0.33–0.97	0.04
	Male	1.00		
BMI	<18.5	1.00		
	18.5–22.9	0.98	0.92–1.05	0.65
	23–24.9	0.48	0.21–1.08	0.04
	≥25	0.46	0.19–1.11	0.08
Incontinence	Yes	0.00	0–1.00	0.98
	No	1.00		
Hypertension	Yes	0.73	0.39–1.36	0.32
	No	1.00		
Hyperlipidemia	Yes	1.38	0.5–3.81	0.54
	No	1.00		
Diabetes	Yes	1.15	0.51–2.60	0.73
	No	1.00		
Surgical site	Trunk	0.00	0–1.00	0.98
	Vertebra	0.64	0.21–1.99	0.44
	Pelvic bone	9.00	1.2–67.42	0.03
	Long bone	1.62	0.48–5.43	0.43
	Joint	1.00		
Preoperative skin condition	Normal	0.85	0.46–1.57	0.59
	Wet/erythema/Philippines thin	10.00	0–15.00	0.99
	Essence/edema/blossom	1.00		
Preoperative limb activity	Partial restriction	0.84	0.48–1.47	0.54
	Completely restricted	3.47	1.43–8.39	0.01
	Unlimited	1.00		
Serum albumin level	High	0.60	0.35–1.02	0.06
	Low	1.00		
Preoperative corticosteroids	Yes	1.70	0.54–5.39	0.37
	No	1.00		
Fasting time	≥20.25 h	3.68	0.6–22.44	0.16
	<20.25 h	1.00		

**TABLE 4:** Univariate logistic regression analysis of the correlation between intraoperative characteristics and IAPI.

Risk factor	Subgroup	OR	95%CI	*p*
Duration of surgery	≥4.75 h	5.71	3.28–9.95	<0.001
	<4.75 h	1.00		
Intraoperative wetting of the compressed part	Yes	2.39	1.21–4.74	0.01
	No	1.00		
Surgical site	Prone position	0.40	0.19–0.84	0.02
	Supine position	0.49	0.21–1.15	0.10
	Beach chair	1.29	0.11–15.6	0.84
	Side-lying position	1.00		
Intraoperative use of the arch frame	Yes	0.53	0.29–0.97	0.04
	No	1.00		
Intraoperative use of head rest and head ring	Yes	0.52	0.29–0.95	0.03
	No	1.00		
Mechanical ventilation	Yes	0.79	0.31–2.00	0.62
	No	1.00		
Intraoperative use of sponge pads/soft pillows	Yes	0.86	0.51–1.46	0.59
	No	1.00		
Intraoperative use of fluid pads	Yes	0.91	0.54–1.55	0.74
	No	1.00		
Intraoperative use of gel pads	Yes	0.85	0.45–1.60	0.61
	No	1.00		
Intraoperative use of foam dressings	Yes	0.00	0–1.00	0.99
	No	1.00		
ASA grade	2	0.00	0–1.00	0.99
	3	0.00	0–1.00	0.99
	4	0.00	0–1.00	0.99
	1	1.00		
Intraoperative use of traction beds	Yes	0.98	0.39–2.44	0.96
	No	1.00		
Intraoperative use of tourniquets	Yes	2.49	0.74–8.33	0.14
	No	1.00		
Intraoperative use of internal fixation devices	Yes	0.44	0.16–1.18	0.10
	No	1.00		
Intraoperative surgical bed adjustment angle	Yes	3.15	1.34–7.41	0.01
	No	1.00		

The risk factors were included in the logistic multifactorial regression analysis according to the criteria of *p* < 0.05. The results of the multifactorial analysis showed that preoperative activity was completely limited (OR = 4.33, 95%CI: 1.41–13.21, *p* = 0.01), operative time ≥4.75 h (OR = 7.58, 95%CI: 3.86–15.50, *p* < 0.001), and intraoperative pressure partial immersion (OR = 2.78, 95%CI: 1.10–6.53, *p* = 0.03) was an independent risk factor for the development of IAPI (Table 5).

**TABLE 5:** Multivariate logistic regression analysis of the correlation between risk factors and IAPI.

Risk factor	Subgroup	OR	95%CI	*p*
Age	<40	1.00		
	40–65	0.99	0.47–2.15	0.99
	66–80	0.39	0.14–0.99	0.054
	>80	0.88	0.15–4.54	0.89
Gender	Female	0.71	0.38–1.35	0.30
	Male	1.00		
BMI	<18.5	1.00		
	18.5–22.9	0.91	0.34–2.55	0.86
	23–24.9	0.79	0.26–2.45	0.68
	≥25	1.24	0.45–3.63	0.68
	<18.5	1.00		
Surgical site	Trunk	0.01	0.01–27.38	0.98
	Vertebra	0.29	0.079–1.25	0.81
	Pelvic bone	2.16	0.19–30.91	0.54
	Long bone	1.18	0.31–5.23	0.81
	Joint	1.00		
Preoperative limb activity	Partial restriction	0.99	0.48–2.02	0.97
	Completely restricted	4.33	1.41–13.20	0.01
	Unlimited	1.00		
Duration of surgery	≥4.75 h	7.58	3.86–15.50	<0.001
	<4.75 h	1.00		
Intraoperative wetting of the compressed part	Yes	2.78	1.10–6.86	0.03
	No	1.00		
Intraoperative use of the arch frame	Yes	3.07	7.95–10.00	0.99
	No	1.00		
Intraoperative use of head rest and head ring	Yes	6.17	0–3.18	0.99
	No	1.00		
Intraoperative surgical bed adjustment angle	Yes	1.58	0.36–6.59	0.53
	No	1.00		

### Establishment of a predictive model for intraoperative acquired pressure injury in orthopedic surgery

**FIGURE 2:**
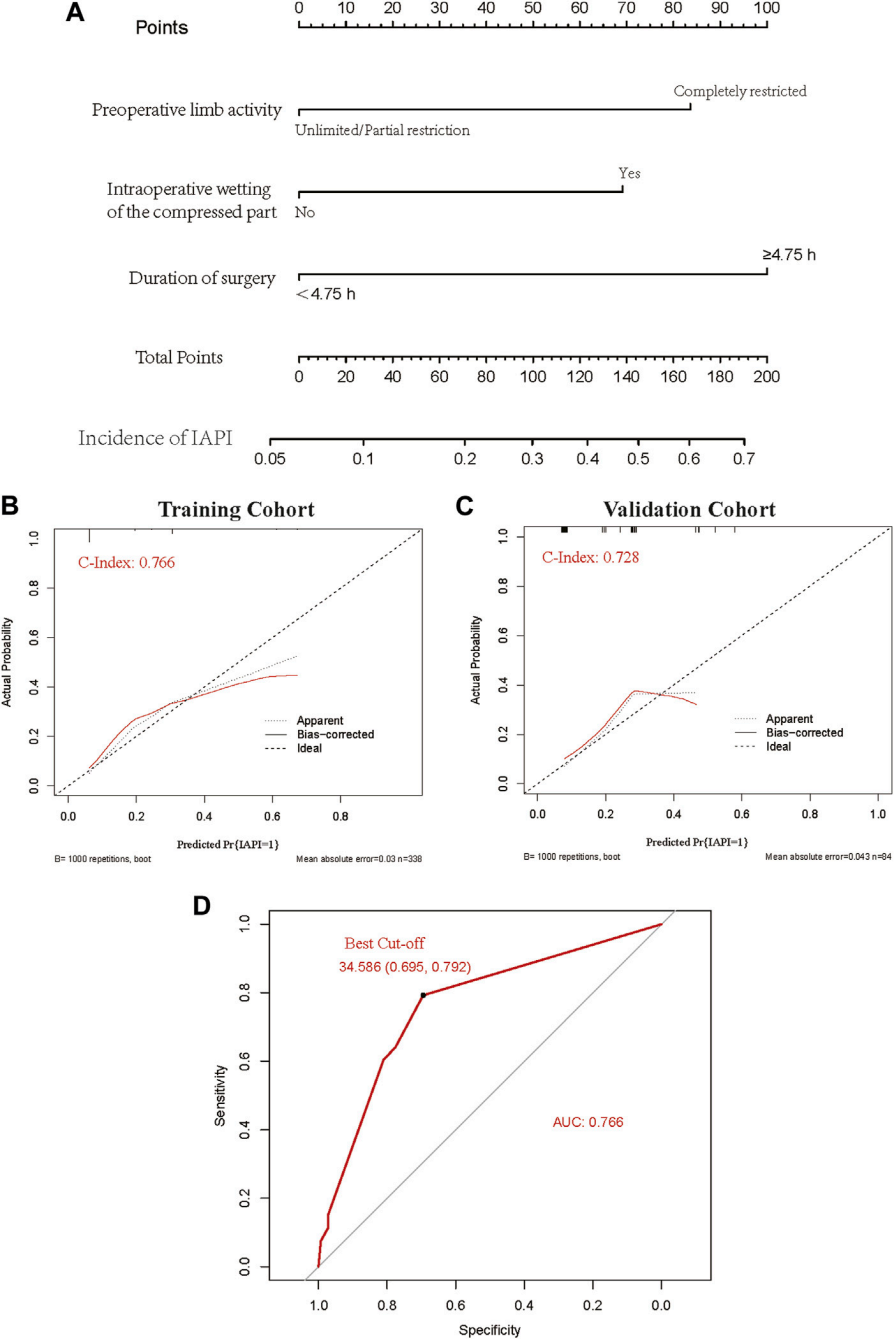
A predictive model was developed for intraoperative acquired pressure injury in orthopedic surgery. **(A)** Nomogram clinical prediction model. **(B)** Calibration of the training dataset. **(C)** Calibration of the validation dataset. **(D)** ROC curve and the best cut-off value.

All included patients (n = 422) were categorized chronologically into training and validation sets in a ratio of 7:3 (338 and 84 cases, respectively). The statistically significant risk factors in the multivariate logistic analysis were used as predictors to establish the nomogram scoring system. The included predictors were preoperative activity limitation, intraoperative wetting of the compressed tissue, and the time of surgery. The score was 87.50 if the preoperative activity was completely restricted and 0 otherwise; the score was 67.50 if the intraoperative compressed tissue was wet and 0 otherwise; the score was 97.50 if the operation time was ≥4.75 h and 0 otherwise. The consistency index of the nomogram scoring system for predicting the probability of pressure injury in the training set was 0.77, with a mean absolute error of 0.033. The consistency index of the nomogram scoring system for predicting the probability of pressure injury in the validation set was 0.73, with a mean absolute error of 0.043. Furthermore, using the ROC curve, the optimal score cut-off value was found to be 34.59 points. All patients with scores above 34.59 should have interventions to prevent the occurrence of IAPI (Figure 2).

## Discussion

In this study, we included 28 orthopedic intraoperative PI risk factors from previous studies and clinical practice. A total of 422 real-world cases were also included, and three independent risk factors—preoperative limb activity, intraoperative wetting of the compressed tissue, and duration of surgery—were successfully identified using chi-squared tests and logistic regression. Finally, the three independent risk factors were successfully used to construct a nomogram clinical prediction model with good predictive validity (area under the ROC curve = 0.77), which is expected to benefit clinical patients.

The literature search and clinical practice suggested 28 risk factors for intraoperative PI in orthopedics; however, the only independent risk factors were preoperative limb mobility, intraoperative presence or absence of wetting of the compressed tissue, and duration of surgery. Previous findings (Reddy, 2011; Gillespie et al., 2021) suggest that patients with poor preoperative mobility, as well as patients who underwent orthopedic surgery with preoperative perceptual limitations, are more likely to have IAPI, which is consistent with our findings. The longer the duration of surgery, the longer the ischemic and hypoperfused state of the compressed tissue; some studies (Gefen et al., 2020) have observed that when the surgery exceeds 4 h, it inevitably causes local skin pressure damage. According to our study, when the surgery time exceeds 4.75 h, it is an independent risk factor, which is generally consistent with the previous findings. Intraoperative wetting of the compressed part is a new risk factor identified in this study from a summary of clinical practice observations and has not been reported previously.

Risk factors such as age, gender, BMI, history of hypertension, surgical site, intraoperative use of the arch frame, intraoperative use of head rest and head ring, and intraoperative surgical bed adjustment angle were statistically significant in chi-squared tests or logistic one-way regression analysis but not in multivariate logistic regression analysis, suggesting that these may be risk factors due to confounding factors and are not independent risk factors.

Our study also included numerous interventions to reduce pressure-related injury, but unfortunately, factors independently influencing PI were not screened. However, the data for all interventions showed a role as protective factors. Therefore, this study suggests that a single measure is not sufficient for the prevention of IAPI in orthopedic surgery patients and that a combination of measures may be needed to effectively prevent PIs. Furthermore, the current methods to prevent pressure-related injuries are limited, and the development of new protective materials is one of the directions of future research.

The Munro Perioperative Pressure Injury Assessment Scale is recommended by the American Association of periOperative Registered Nurses for assessing the risk of PI before, during, and after surgery (Munro, 2010). The Chinese version of the Munro Pressure Injury Risk Assessment Scale has often been compared with the Braden scale (Huang et al., 2021; Lei et al., 2022), Norton scale (Kwong et al., 2005), and Waterlow scale (Luo et al., 2021), and the results showed that the Munro Pressure Injury Risk Assessment Scale was superior for assessing surgical patients but not widely used because of its complex score of entry and cumbersome assessment. Currently, all major hospitals have their risk scales based on the results of previous studies and, to a great extent, have been able to effectively reduce the incidence of high-grade stress injuries. However, the prediction and prevention abilities of low-grade PI are insufficient, and there is still a risk of postoperative development of severe PI. Except for the scale, machine learning and integrated data based on algorithms to detect individual PI risk and adopt prevention strategies have achieved satisfactory success as well. Song et al. (2021) found that the random forest model performed best and achieved a high AUC in predicting PI. Gojiro et al. (Nakagami et al., 2021) confirmed that the XGBoost model achieved the highest sensitivity and AUC. Many other machine learning models have been developed in predicting PI (Hu et al., 2020; Zhou et al., 2023). However, there are complex codes in the machine learning model, which could not be used in clinical practice easily. Developing some software based on the machine learning model may address this issue. On the contrary, nomograms can be used in clinical practice while being extremely convenient. Although many nomogram models have been developed in predicting PI (Corniello et al., 2014; Moyse et al., 2017; Gao et al., 2018), the nomogram model for IAPI has not been developed yet. Accordingly, our model just addresses this critical issue. Meanwhile, Gao et al. (2018) used the logistic regression model to develop a risk assessment of intraoperatively acquired pressure injury, which contained patients who underwent neurosurgery, orthopedics, pediatric surgery, and cardiac surgery therapy. Their model included five risk factors: applied external force, cardiopulmonary bypass, thinness, operation duration, and intraoperative blood loss, which are consistent with our analysis results, especially the operation duration, in addition to identifying two new risk factors.

The results of our prediction model show that all three screened risk factors scored above the optimal cut-off point, suggesting that interventions should be initiated whenever a risk factor is present and that interventions should become more refined as the risk score increases. These three factors are essentially different from those noted in previous scales, and therefore, in clinical practice, they can be assessed using our model on the basis of the original scales, enabling improved early identification and intervention to eliminate the occurrence of stress injuries. Subsequent prospective studies in large cohorts can improve our model.

Our study also has limitations. Due to ethical requirements and the primary principle of patient benefit, all patients in the control group had undergone interventions using our own established scales, which inevitably had some bias. For example, previous studies suggested that BMI was a significant risk factor for PI but was not statistically significant in this study, possibly because all patients had already received the first intervention, which greatly reduced the incidence of PI, while factors such as BMI were important reference data for these scales, leading to an unavoidable bias. The age and BMI could not show significant differences in the multivariate logistic regression analysis; other reasons may be that the cut-off value is not accurate, so the development of appropriate statistical analysis methods to determine the optimal cut-off value is needed. At the same time, the first scale assessment prevented the full accuracy of some interventions and led to uncontrolled bias. Nonetheless, the presence of the first intervention allowed us to identify new factors independent of the scale and provided new ideas and methods for preventing PI. The next step to optimize the prediction model might be an inclusion of missing risk factors that are relevant but not yet documented regularly. Automatically extracted risk factors from clinical records might help increase data collection and develop big data based on models. To summarize, this model can help optimize prevention strategies in clinical practice, but further investigations are needed.

## Conclusion

In conclusion, we successfully identified new independent risk factors for intraoperative PI-related injuries in orthopedic patients and developed a clinical prediction model to serve as an important complement to existing scales and provide additional benefits to patients. Our study suggests that a single measure is not sufficient for the prevention of IAPI in orthopedic surgery patients and that a combination of measures may be required for the effective prevention of IAPI.

## Data Availability

The raw data supporting the conclusion of this article will be made available by the authors, without undue reservation.

## Supplementary material

SUPPLEMENTARY FIGURE S1 PRISMA of systematic review.

SUPPLEMENTARY FIGURE S2 Flow chart of prediction model development.

SUPPLEMENTARY FIGURE S3 Pictures of IAPI.
