# Supplementary figures and images for: The prediction model for intraoperatively acquired pressure injuries in orthopedics based on the new risk factors: a real-world prospective observational, cross-sectional study
Source: Front Physiol. 2023 Jul 21;14:1170564. doi: 10.3389/fphys.2023.1170564 (PMC10401272; doi:10.3389/fphys.2023.1170564)

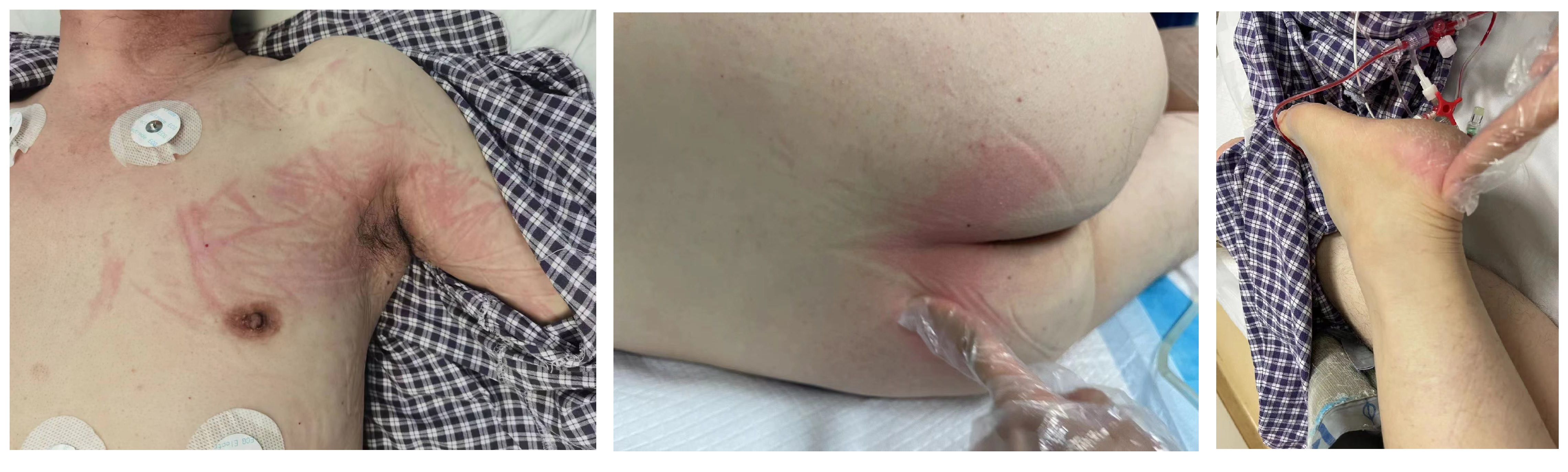

Supplement: Supplementary file 1 [file Image3.JPEG]

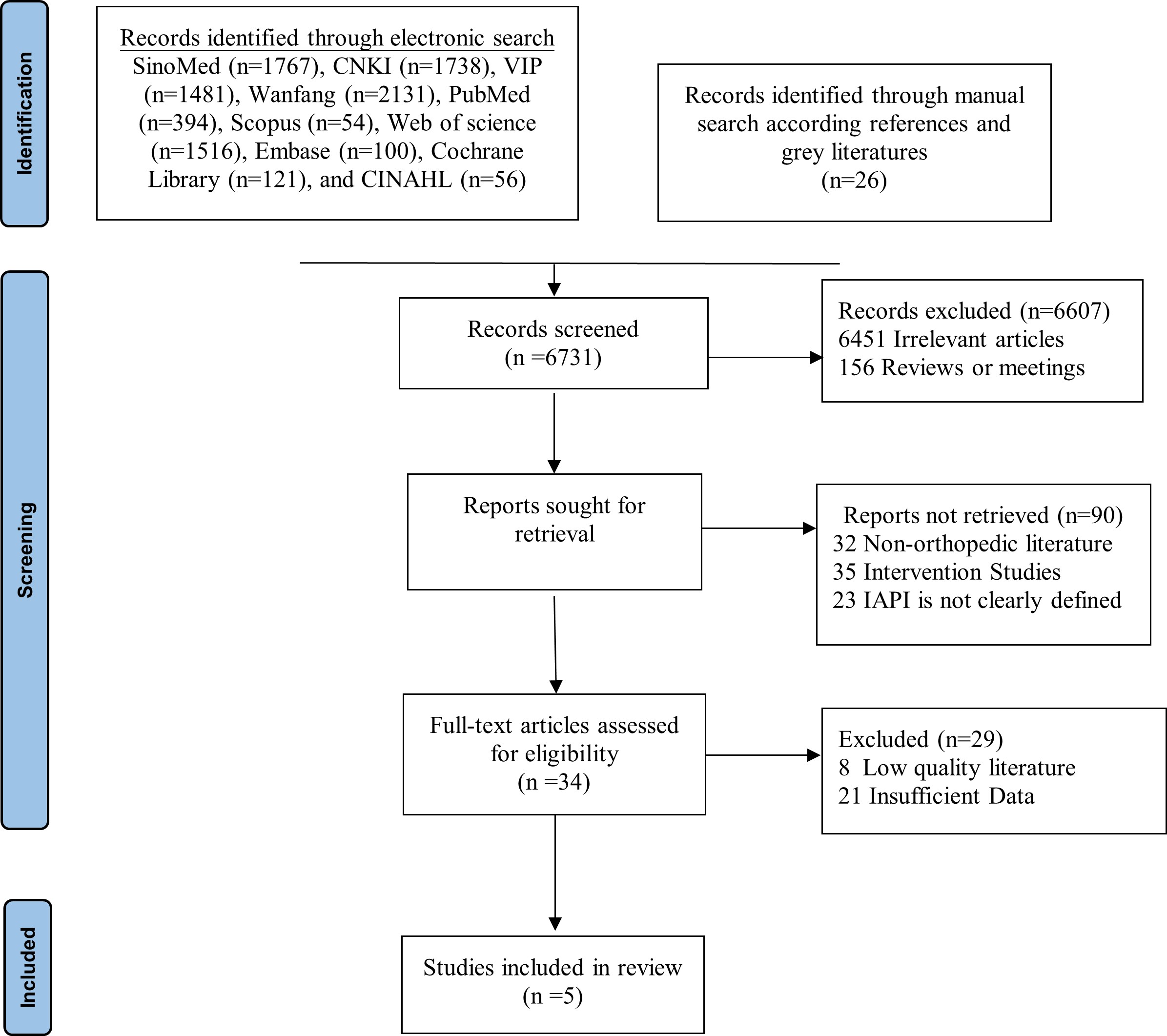

Supplement: Supplementary file 2 [file Image1.JPEG]

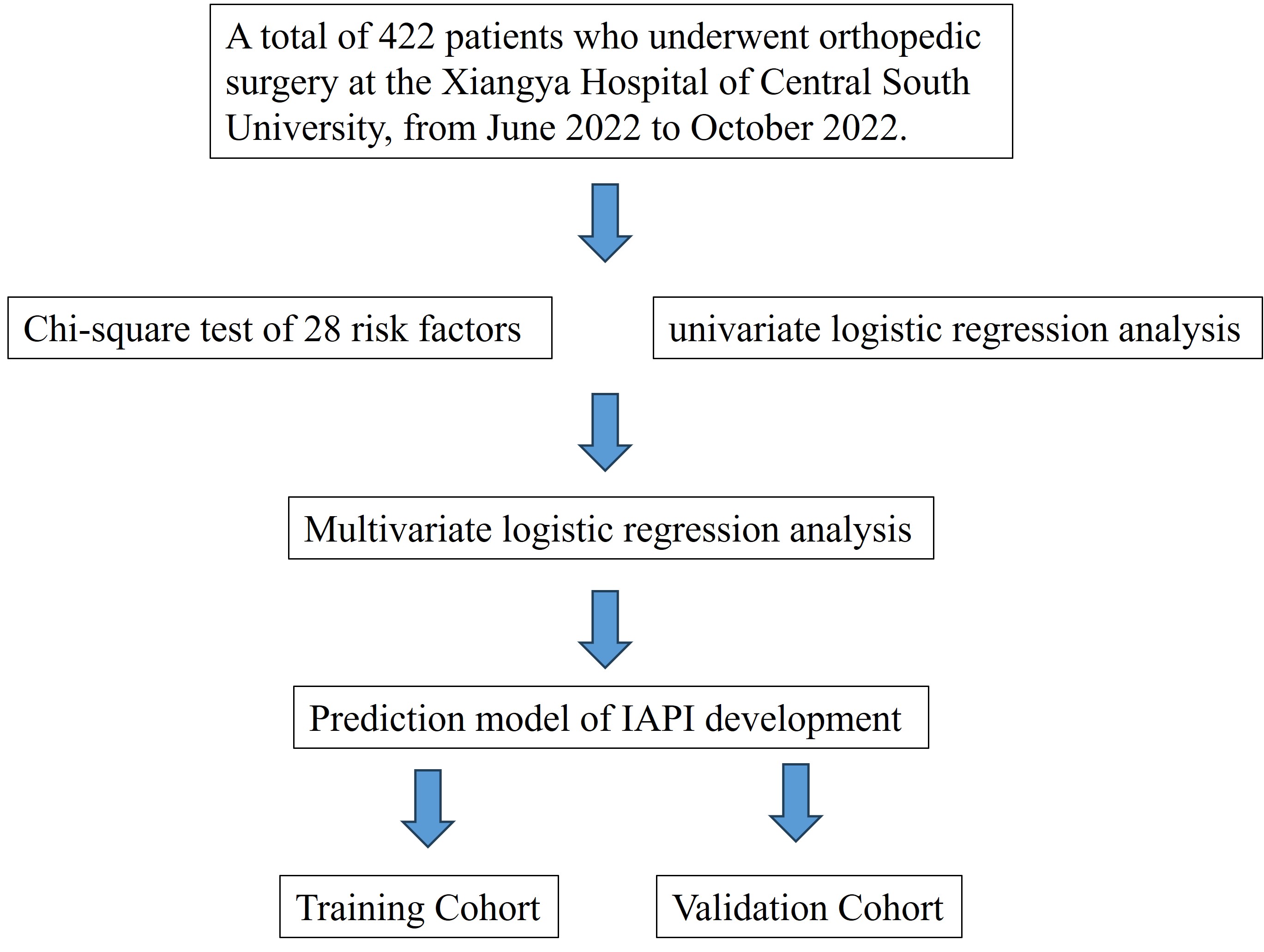

Supplement: Supplementary file 3 [file Image2.JPEG]
